# A preclinical model of patient-derived cerebrospinal fluid circulating tumor cells for experimental therapeutics in leptomeningeal disease from melanoma

**DOI:** 10.1093/neuonc/noac054

**Published:** 2022-02-25

**Authors:** Vincent Law, Zhihua Chen, Francesca Vena, Inna Smalley, Robert Macaulay, Brittany R Evernden, Nam Tran, Yolanda Pina, John Puskas, Gisela Caceres, Simon Bayle, Joseph Johnson, James K C Liu, Arnold Etame, Michael Vogelbaum, Paulo Rodriguez, Derek Duckett, Brian Czerniecki, Ann Chen, Keiran S M Smalley, Peter A Forsyth

**Affiliations:** Department of Tumor Biology, H. Lee Moffitt Cancer Center & Research Institute, Tampa, Florida, USA; Department of Neuro-Oncology, H. Lee Moffitt Cancer Center & Research Institute, Tampa, Florida, USA; Department of Biostatistics and Bioinformatics, H. Lee Moffitt Cancer Center & Research Institute, Tampa, Florida, USA; Department of Drug Discovery, H. Lee Moffitt Cancer Center & Research Institute, Tampa, Florida, USA; Department of Cancer Physiology, H. Lee Moffitt Cancer Center & Research Institute, Tampa, Florida, USA; Department of Pathology, H. Lee Moffitt Cancer Center & Research Institute, Tampa, Florida, USA; Department of Analytic Microscopy, H. Lee Moffitt Cancer Center & Research Institute, Tampa, Florida, USA; Department of Analytic Microscopy, H. Lee Moffitt Cancer Center & Research Institute, Tampa, Florida, USA; Department of Tumor Biology, H. Lee Moffitt Cancer Center & Research Institute, Tampa, Florida, USA; Department of Analytic Microscopy, H. Lee Moffitt Cancer Center & Research Institute, Tampa, Florida, USA; Department of Pathology, H. Lee Moffitt Cancer Center & Research Institute, Tampa, Florida, USA; Department of Pathology, H. Lee Moffitt Cancer Center & Research Institute, Tampa, Florida, USA; Department of Drug Discovery, H. Lee Moffitt Cancer Center & Research Institute, Tampa, Florida, USA; Department of Analytic Microscopy, H. Lee Moffitt Cancer Center & Research Institute, Tampa, Florida, USA; Department of Neuro-Oncology, H. Lee Moffitt Cancer Center & Research Institute, Tampa, Florida, USA; Department of Neuro-Oncology, H. Lee Moffitt Cancer Center & Research Institute, Tampa, Florida, USA; Department of Neuro-Oncology, H. Lee Moffitt Cancer Center & Research Institute, Tampa, Florida, USA; Department of Immunology, H. Lee Moffitt Cancer Center & Research Institute, Tampa, Florida, USA; Department of Drug Discovery, H. Lee Moffitt Cancer Center & Research Institute, Tampa, Florida, USA; Department of Breast Oncology, H. Lee Moffitt Cancer Center & Research Institute, Tampa, Florida, USA; Department of Biostatistics and Bioinformatics, H. Lee Moffitt Cancer Center & Research Institute, Tampa, Florida, USA; Department of Tumor Biology, H. Lee Moffitt Cancer Center & Research Institute, Tampa, Florida, USA; Department of Tumor Biology, H. Lee Moffitt Cancer Center & Research Institute, Tampa, Florida, USA; Department of Neuro-Oncology, H. Lee Moffitt Cancer Center & Research Institute, Tampa, Florida, USA

**Keywords:** ceritinib, leptomeningeal disease (LMD), melanoma, patient-derived CSF-CTCs (PD-CSF-CTCs), single-cell RNA sequencing

## Abstract

**Background:**

Leptomeningeal disease (LMD) occurs as a late complication of several human cancers and has no rationally designed treatment options. A major barrier to developing effective therapies for LMD is the lack of cell-based or preclinical models that recapitulate human disease. Here, we describe the development of *in vitro* and *in vivo* cultures of patient-derived cerebrospinal fluid circulating tumor cells (PD-CSF-CTCs) from patients with melanoma as a preclinical model to identify exploitable vulnerabilities in melanoma LMD.

**Methods:**

CSF-CTCs were collected from melanoma patients with melanoma-derived LMD and cultured *ex vivo* using human meningeal cell-conditioned media. Using immunoassays and RNA-sequencing analyses of PD-CSF-CTCs, molecular signaling pathways were examined and new therapeutic targets were tested for efficacy in PD-CSF-CTCs preclinical models.

**Results:**

PD-CSF-CTCs were successfully established both *in vitro* and *in vivo*. Global RNA analyses of PD-CSF-CTCs revealed several therapeutically tractable targets. These studies complimented our prior proteomic studies highlighting IGF1 signaling as a potential target in LMD. As a proof of concept, combining treatment of ceritinib and trametinib *in vitro* and *in vivo* demonstrated synergistic antitumor activity in PD-CSF-CTCs and BRAF inhibitor-resistant melanoma cells.

**Conclusions:**

This study demonstrates that CSF-CTCs can be grown *in vitro* and *in vivo* from some melanoma patients with LMD and used as preclinical models. These models retained melanoma expression patterns and had signaling pathways that are therapeutically targetable. These novel models/reagents may be useful in developing rationally designed treatments for LMD.

**Key Points**

- For the first time, we propagated CSF-CTCs from LMD patients in vitro and in vivo.
- We identified therapeutically relevant target using scRNA-seq for patient-derived CSF-CTCs.
- PD-CSF-CTCs may be valuable to better understanding LMD biology and in rational drug design for this disease.

**Importance of the Study**

LMD is a devastating complication of several cancers with very short survival and without effective therapies. A major barrier to the development of rational drug design strategies for LMD has been the inability to propagate PD-CSF-CTCs *in vitro* and *in vivo*. Here, we describe the successful culture and expansion of PD-CSF-CTCs from melanoma patients *in vitro*, and *in vivo* using xenograft models. We identified a therapeutically tractable target (IGF1R) whose pharmacological inhibition prolonged survival using *in vivo* PD-CSF-CTC models of LMD. We anticipate that these reagents/models will lead to the development of rational drug design strategies for LMD.

A complication of advanced melanoma is leptomeningeal disease (LMD), the development of tumor cells in the central nervous system (CNS) and cerebrospinal fluid (CSF).^1^ Patients with LMD have a dismal prognosis, with survival ranging from weeks to months,^1–3^ and no truly effective treatments exist.

Recent studies have shown how cancer cells colonize the unique environment of the leptomeningeal space,^4,5^ and we have previously performed unbiased *omics-based* analyses of CSF from patients with LMD.^2^ We found that the CSF was enriched with proteins involved in innate immunity, protease-mediated damage, and insulin-like growth factor– (IGF–) related signaling. Similarly, our single-cell analyses of LMD showed specific upregulation of several immunocyte subtypes in the CSF that were distinct from immunocyte populations in brain metastases or systemic metastases.^6^

CSF circulating tumor cells (CSF-CTCs) from liquid biopsies are useful for genetic profiling and molecular characterization of LMD, but the scarcity of these cells in CSF poses an obstacle to performing experiments that are critical for better understanding LMD biology and therapeutic development. There have been no reports of the successful propagation of patient-derived CSF-CTCs (PD-CSF-CTCs) from melanoma patients. This may reflect the challenges of growing these rare and fragile cells and the difficulty of adequately reproducing the CSF microenvironment. Human peripheral blood (PB) CTCs were successfully inoculated *in vivo* from several cancers,^7,8^ and these preclinical models resembled human disease, retaining therapeutically relevant targets and making them highly valuable for drug discovery.^9^ To our knowledge, breast cancers are the only source of CSF-CTCs that have been cultured *in vitro*,^10^ and there are no reports of *in vivo* LMD models derived from PD-CSF-CTCs. Here we describe, for the first time, the isolation of melanoma CSF-CTCs from patients for *in vitro* propagation and *in vivo* LMD model development. For the first time, we successfully generated PD-CSF-CTC cultures via *in vitro* and *in vivo* expansions. The ability to grow CSF-CTCs will be a valuable asset for future development of rationally designed LMD therapies.

## Materials and Methods

### Patient Specimen Collection and Processing

Our deidentified patient specimen collection protocol was approved by the University of South Florida’s Institutional Review Board (MCC 50103, 50172, and 19332). CSF was collected from various site/time points, including from lumbar puncture (LP), during an Ommaya reservoir placement surgery, from an Ommaya reservoir, and during an autopsy. CSF was immediately placed on ice and then CSF and cell pellets were separated by centrifugation at 1500 rpm for 5 minutes at 4° C. CSF was aliquoted and stored frozen, whereas cell pellets were either resuspended in human meningeal cell– (HMC–) conditioned media for culturing or cryopreserved. Patient and melanoma characteristics were also collected.

### CellSearch for CSF-CTC Enumeration

Complete methods for this procedure are detailed in Supplementary Methods. In brief, CTCs in CSF from patients with LMD were detected by adapting the CellSearch system (Janssen Diagnostics, Raitan, NJ, USA) using the CELLTRACKS Circulating Melanoma Cell Kit as previously described.^11^

### HMC, CSF-CTC, and Melanoma Cell Line Culturing

HMCs were purchased from ScienCell Laboratories (Carlsbad, CA, USA) and maintained in poly-L-lysine–coated T175 culture flasks containing complete Meningeal Cell Medium (MenCM) (MenCM + meningeal growth factors + 2% fetal bovine serum (FBS) + penicillin/streptomycin solution [all from ScienCell Laboratories, Carlsbad, CA, USA]). When cells reached 70% confluence, the media was centrifuged at 1500 rpm for 5 minutes, supernatant was collected for generating HMC-conditioned media to culture CSF-CTCs. Cell pellets from CSF was resuspended in a 1:1 ratio of HMC-conditioned media to complete MenCM, with additional 40 ng/ml of fibroblast growth factor (FGF) and 40 ng/ml of epidermal growth factor (EGF) (both Stemcell Technologies, Vancouver, BC, Canada). CSF-CTCs were cultured in single wells of a 96-well plate until confluent, and then transferred to larger culturing apparatus. Culture media was refreshed every 3 days. Melanoma cell line cultures details are in Supplementary Methods.

### Immunofluorescence Assays

Cells were fixed using 4% paraformaldehyde on an 8-well chamber glass slide (Thermo Fisher Scientific, Alachua, FL, USA). Complete immunostaining procedure and computer-assisted analyses are described in Supplementary Methods.

### RNA Sequencing Characterization of PD-CSF-CTCs

Single-cell RNA sequencing and analysis techniques have been described in detail by our group.^6,12^ To facilitate rapid analysis of single-cell datasets in a user-friendly manner, the Interactive Single Cell Visual Analytics (ISCVA) tool, which we developed, was used.^6^ The webtool is accessible to public at http://iscva.moffitt.org. Details on quality control/cell typing methodology can be found in Supplementary Methods.

### In Vitro Drug Efficacy and Combination Assays

Twenty-five microliters of 2.5 × 10^4^ cells/ml suspension were seeded in a 384-well plate (Greiner Bio-One, Kremsmünster, Austria) and treated for 72 hours with increasing concentrations of ceritinib (Chemie Tek, Indianapolis, IN, USA) or trametinib (Chemie Tek, Indianapolis, IN, USA).^13^ Inhibition of proliferation was measured by the CellTiter-Glo Luminescent Cell Viability Assay (Promega, Madison, WI, USA). Drug synergism or antagonism was determined using Compusyn software to calculate the combination index (CI) values using the Chou-Talalay method.^14^ A CI < 1 was considered synergistic, CI > 1 was antagonistic, and CI = 1 was additive. All data represent the mean of 3 independent experiments.

### In Vivo LMD Xenograft and Patient-Derived Xenograft Models

Cancer cells (PD-CSF-CTCs, WM164, and WM164R) were virally transduced with enhanced GFP–NanoLuc plasmid. To generate the LMD xenograft models, microsurgery was performed to inject 5.0 × 10^4^ enhanced NanoLuc–labeled cancer cells into the CSF space via cisterna magna of 6- to- 8-week-old NOD scid gamma (NSG) mice (The Jackson Laboratory, Bar Harbor, ME, USA) as previously described.^15^ Status of LMD was assessed once a week. Bioluminescence signals were detected by intraperitoneally injecting 0.1 cc of a 1:40 dilution of NanoLuc-reporter substrate (Promega, Madison, WI, USA) in sterile phosphate-buffered saline into the animal. Bioluminescence images (BLI) were captured using the Xenogen IVIS 200 system (Xenogen, Alameda, CA, USA), and BLI analyses were performed using Living Image Software (PerkinElmer, Waltham, MA, USA). Treatment for LMD began seven to fourteen days postinjection of cancer cells via the cisterna magna (or when LMD was first detected via BLI), mice were given 10 mg of compounds/kilogram of ceritinib (Chemietek, Indianapolis, IN, USA), 1 mg of compounds/kilogram of trametinib (Chemietek), or both once a day via oral gavage; treatment lasted for 49 days (7 weeks). To sample CSF from xenograft mice under anesthesia, an incision approximately 1 cm long was made between the skull and C2 vertebra. A 30 g needle was inserted into the cisterna magna and CSF was withdrawn. Mice were subsequently euthanized. Institutional animal care and use committee approval was obtained from the University of South Florida (IS00005974).

### Human Receptor Tyrosine Kinase Phosphorylation and Human Growth Factor Profiling

Human Phospho-Receptor-Tyrosine-Kinase Array (R&D Systems, Minneapolis, MN, USA) and Human-Growth-Factor Array CI (Raybiotech, Peachtree Corners, GA) kits were used according to the manufacturer’s instructions. The method to quantify the immunoblot is described in Supplementary Methods.

### Histopathologic Slide Assessment

Histologic slides stained with hematoxylin and eosin were assessed by the pathologist, Dr. Robert Macaulay. The histopathologic assessment is described in Supplementary Methods.

### Statistics

Bar graph results were reported as mean values, with error bars indicating ± standard error of the mean. The magnitude of changes between different conditions was determined using parametric paired *t* test. Kaplan-Meier survival curves were constructed, and the Mantel-Cox test was used to determine significant differences between cohorts. GraphPad Prism 6 software was used to calculate statistical significance.

## Results

### Collection of CTCs From the CSF of Patients with LMD From Melanoma

CSF specimens from 11 melanoma patients with LMD were collected during surgery, via LP, via Ommaya reservoirs, or during rapid autopsy (Table 1). All patients except for 3 (patients 13, 15, and 18) had *BRAF V600E*-mutated melanoma. The median overall survival after the diagnosis of LMD was 2.7 months (range, 0.7–29 months) with all patients succumbing to LMD. The amount of CSF collected varied depending on clinical availability/patient tolerance and whether the patient consented for autopsy. For patients who had multiple CSF collections, we enumerated CSF-CTCs from the first visit. The median number of CTCs per ml was 21.2 (range, 0.13–2133.6 CSF-CTCs/ml).

**Table 1.** Clinical and Cerebrospinal Fluid (CSF) Characteristics of Melanoma-Associated LMD Patients

ID	Age	Life status	Location initial melanoma	Mutations	Cytology	Survival (Days)	Type of treatments received prior to development of LMD	Number of CSF collections from Ommaya, LP, or surgery	Cell Search CSF-CTCs (per ml CSF)	Were CSF-CTCs able to grow *in vitro*? (1 = yes, 0 = no)	Were CSF-CTCs able to expand for more than 6 months? (1 = yes, 0 = no)
#8	36	Dead	Right shoulder	BRAF V600E	Suspicious.	869	none	6	19.4	0	0
#9	53	Dead	Right cheek spitz nevus	BRAF V600E	+	127	pembro	6	2133.6	1	1
#10	36	Dead	Right chest	BRAF V600E	Atypical	167	TIL, vemu, IL2, dab/tra, crani + FSRT for BM, Ipi/nivo	8	7.3	0	0
#11	39	Dead	Right back	BRAF V600E	+	63	crani + FSRT + SRS for BM, ipi/nivo, WBRT for BM, dab/tra, repeat WBRT + SRS for BM,	1	nd	1	0
#12	69	Dead	Low back	BRAF V600E	+	31	nivo, dab/tra	1	nd	1	1
#13	67	Dead	Scalp	NRas	+	68	MAGE vaccine, ipi, pembro, ipi/nivo, ipi	2	0.13	1	0
#14	38	Dead	Abdominal wall skin	BRAF V600E	–	34	crani for BM, FSRT planned	2	22.9	0	0
#15	62	Dead	Abdominal wall skin	BRAF wt	+	81	nivo	2	30.5	1	0
#16	31	Dead	Posterior calf	BRAF V600E	nd	22	ipi, dab/tra, WBRT + TMZ for BM, dab/tra + pembro	0	nd	1	1
#17	60	Dead	Lower back	BRAF V600E	–	228	crani + FSRT for BM	2	0.5	1	0
#18	67	Dead	left leg	BRAFG469E, NF1mt	Suspicious	443	ipi, pembro, crani for intravenricular tumor causing acute hydro	2	0.4	0	0

pembro, pembrolizumab; TIL, tumor infiltrating lymphocytes; vemu, vemurafenib; IL2, interleukin2; dab, dabrafenib; tra, trametinib; ipi, ipilimumab; nivo, nivolumab; WBRT, whole brain radiation therapy; BM, brain metastasis; FSRT, fractionated stereotactic radiotherapy; SRS, stereotactic radiotherapy; TMZ, temozolomide.

### Optimization of Short- and Long-Term Culturing of Melanoma CSF-CTCs From Patients to Generate Patient-Derived Cerebral Spinal Fluid-Circulating Tumor Cells (PD-CSF-CTCs)

**Figure 1.**
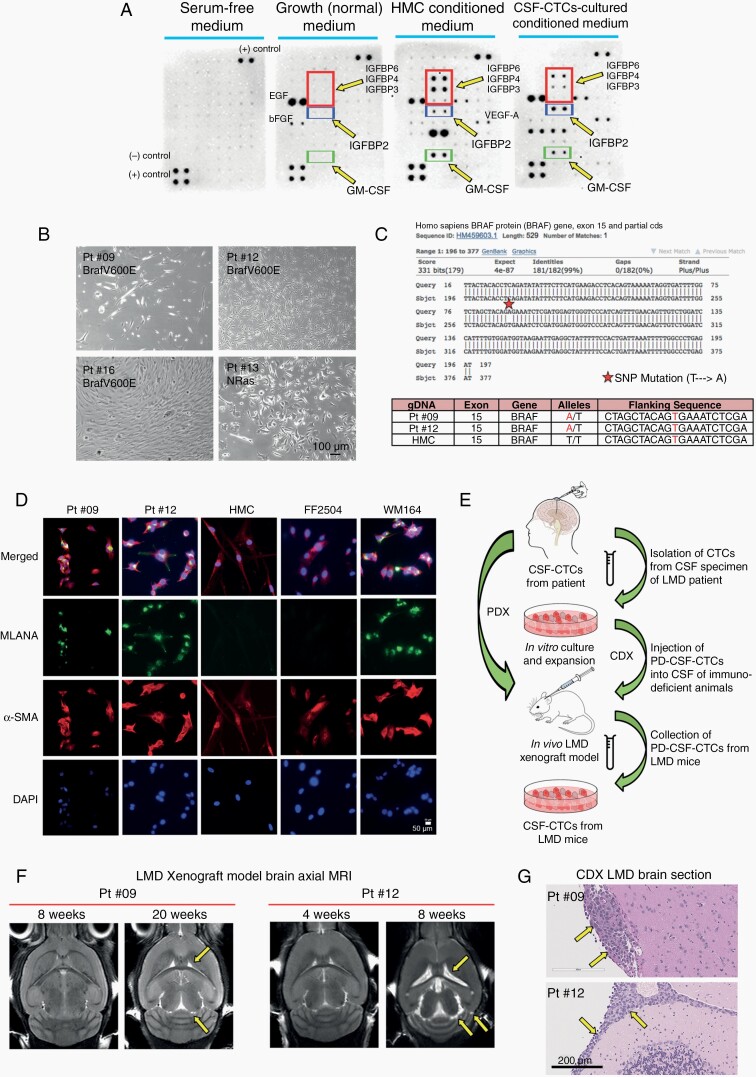
*Ex vivo* culture of melanoma PD-CSF-CTCs and examples of successful *in vivo* culture in LMD mouse model. (A) Immunoblot from a Human-Growth-Factor array comparing serum free medium, normal growth medium, HMC-conditioned medium, and HMC-conditioned medium that was cultured with CSF-CTCs. Arrows are pointing at duplicate blots representing each growth factor. (B) Representative bright field images of propagating *ex vivo* melanoma CSF-CTCs from four patients; three were *BRAF V600E* mutants (patients 9, 12, 16), and one was NRAS mutant (Pt #13). Each CSF-CTC culture displayed distinctive cell morphology. Bar = 100 μm. (C) Mutation status of growing PD-CSF-CTCs from patients 9 and 12 were evaluated by using single-nucleotide polymorphism genotyping. Both melanoma patients were *BRAF V600E*. HMC was used as control. (D) Representative immunofluorescence images showing that PD-CSF-CTCs are melanocytic in origin and expressed Melan-A, and α-SMA, whereas our negative controls (HMCs and human fibroblast cells FF2504) did not. Bar = 50 μm. (E) A schematic of how *ex vivo* CSF-CTCs were expanded. CTCs in CSF were expanded *in vitro* until sufficient cells were available to inoculate into a murine PDX and/or CDX models *in vivo*. CSF-CTCs were collected from LMD mice. (F) Brain MRIs of LMD mice from CDX model showed enlarged ventricles and hydrocephaly (arrows). G) H&E stained brain sections of patients 9 and 12 CDX LMD mice. Cancer cells metastasized in the meninges (arrows). Bar = 200 μm.

We next determined whether *ex vivo* expansion of CSF-CTCs was possible (Table 1) and employed a number of strategies (Supplementary Table 1).^16–18^ Consistently, we found culturing the CTCs in HMC-conditioned media supplemented with FGF and EGF was the most successful with 7 of the 11 (64%) patients’ CTCs expanding *in vitro* (Supplementary Table 1, strategy 8). The HMC-conditioned media contain secreted growth factors, notably IGF-binding proteins (IGFBPs), granulocyte-macrophage colony-stimulating factor (GM-CSF), and vascular endothelial growth factor A (VEGF-A) (Figure 1A). We noted that only adherent CSF-CTCs survived *in vitro* with growth rates. These cells grew slowly, and had varied cell morphologies (Figure 1B). Of the seven successful CSF-CTC cultures, 5 eventually became static after a number of weeks of serial passaging. Remarkably, patients 9 and 12 derived CSF-CTCs continued to proliferate logarithmically and ultimately, could be passaged *in vitro* in normal HMC (non-conditioned) medium. Patient 12’s PD-CSF-CTCs originated from CSF collected during autopsy, whereas patient 9’s PD-CSF-CTCs were obtained from both LP and autopsy. We verified long-term cell cultures were melanoma by confirming the presence of *BRAF V600E* mutation and by staining for Melan-A, a melanocyte marker (Figure 1C&D).

For *in vivo* expansion, we inoculated CSF-CTCs of 4 patients (patients 9, 12, 15, and 16) in the CSF of immunodeficient mice (NSG) (Table 2). We utilized both cell line-derived xenograft (CDX) and patient-derived xenograft (PDX) approaches. For the CDX model, CSF-CTCs were briefly propagated *in vitro* (for patients 9, 12, 16) to at least 5.0 x 10^4^ cells before inoculation, and for the PDX model, we injected the noncultured CTCs from patient’s CSF (for patients 9, 15). (Figure 1E). None of the PDX mice developed LMD, possibly because of a low number of CSF-CTCs available and an unknown cell viability as starting material. In CDX model, mice injected with PD-CSF-CTCs from patients 9 and 12 resulted in LMD: patient 9 mice (*n* = 3) developed LMD at 20 weeks, and patient 12 mice (*n* = 2) at 8 weeks. Disease mice showed >15% weight loss, displayed ataxia (Supplementary Table 2) and had hydrocephalus as identified via MRI (Figure 1F). H&E brain sections confirmed metastasis in the meninges (Figure 1G). We collected CSF-CTCs from CDX mice, and these cells were further expanded *in vitro* or *in vivo*.

**Table 2.** Characteristics of Patient-Derived Cerebrospinal Fluid Circulating Tumor Cells (PD-CSF-CTCs) That Were Attempted for Patient-Derived Xenograft (PDX) and/or Cell Line-Derived Xenograft (CDX) Models

ID	*In vitro* culture success (1 = yes, 0 = no)	CSF-CTCs collection method	PDX model attempted (1 = yes, 0 = no)	Number of PD-CSF-CTCs injected for PDX model	PDX success (1 = yes, 0 = no)	CDX model attempted (1 = yes, 0 = no)	Number of PD-CSF-CTCs injected for CDX model	CDX success (1 = yes, 0 = no)	Number of mice injected	LMD detection method	Number of weeks before LMD developed
#9	1	Ommaya and Autopsy	1	n/a	0	1	50 000	1	4	MRI	20
#12	1	LP and Autopsy	0	n/a	0	1	50 000	1	2	MRI	8
#15	1	Ommaya	1	n/a	0	0	n/a	0	1	MRI	Unsuccessful
#16	1	Autopsy	0	n/a	0	1	50 000	0	4	MRI	Unsuccessful

### The In Vivo LMD Model of PD-CSF-CTCs Resembles LMD CSF-CTCs Among Patients

**Figure 2.**
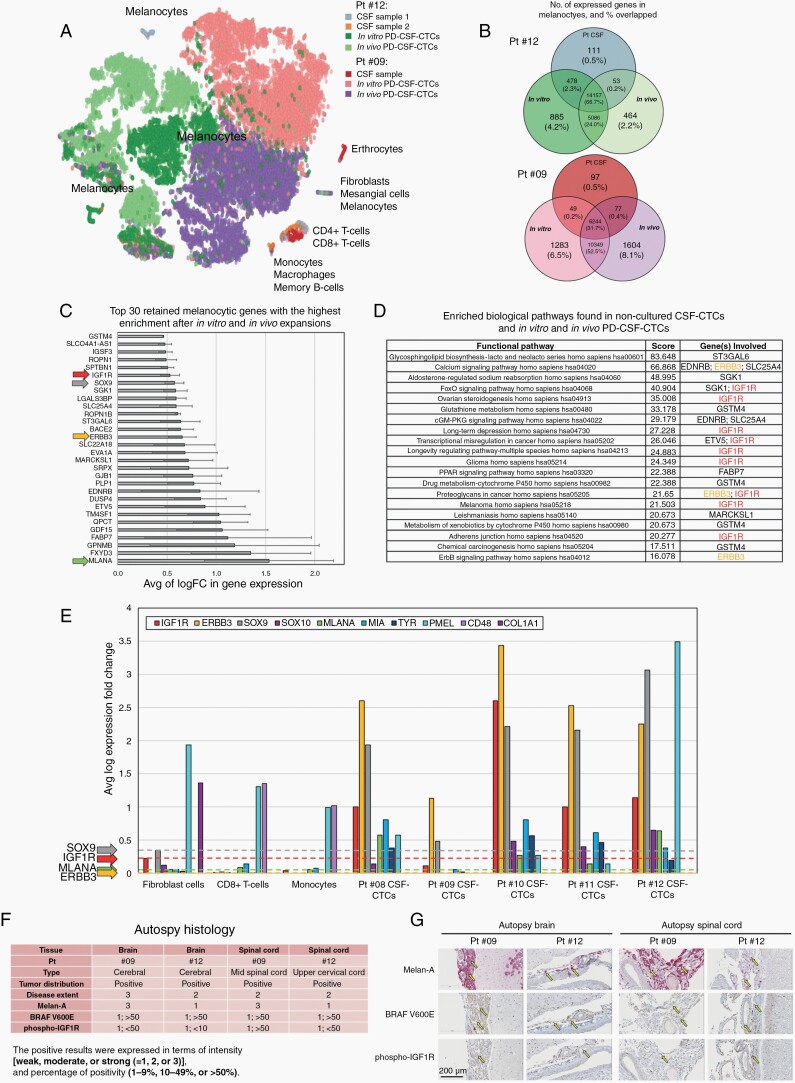
Transcriptome analysis of PD-CSF-CTCs shows adaptation to *ex vivo* culture and retention of cardinal melanoma genes including *IGF1R*, *ErbB3*, and *Sox9.* (A) t-SNE plot showing major cell types identified in patients 9’s and 12’s CSF-CTCs, and their respective *in vitro* and *in vivo* propagated PD-CSF-CTCs. (B) Venn diagrams showing the number of genes expressed that were unique to noncultured CSF-CTCs (Pt CSF) and those that were retained after *in vitro* (*In vitro*) and *in vivo* (*In vivo*) propagations. (C) Graph representing a list of 30 most enriched (logFC > 0.4 in gene expression; *P* < .05) melanoma-associated gene signatures that were retained after *in vitro* and *in vivo* propagations. (D) A list of 20 most enriched biological pathways in accordance to gene signatures in (C), identified by using the KEGG pathway database (E) Average log expression of melanoma-associated gene signatures of non-tumor cells (fibroblasts and immunocytes) in CSF and CSF-CTCs from LMD patients. Comparisons were made between patients 8, 9, 10, 11, and 12. (I) IHC scores of Melan-A, BRAF V600E, and phospho-IGF1R expression in brain and spinal cord tissues from patients 9 and 12, which were obtained from autopsies. (J) Representative autopsy slides of (I); Melan-A, BRAF V600E, and phospho-IGF1R staining by IHC. Bar = 200 μm.

Because of the selective pressure of *in vitro* and *in vivo* expansions, we next assessed how reflective the long-term PD-CSF-CTC cultures were of the original patient-derived cells using scRNA-seq analysis. We targeted a minimum of 5000 cells in each of the *in vitro* and *in vivo* PD-CSF-CTC cultures, and all single cells that were present in the noncultured CSF. For the analyses, we developed a 2-stage architecture analytic tool called ISCVA, through which we could process scRNA-seq data and visualize different subpopulations of cells and genes in Uniform Manifold Approximation and Projection (UMAP) and t-distributed stochastic neighbor embedding (t-SNE) format as described.^6^ We merged the scRNA-seq data of patients 9’s and 12’s CSF samples and their respective *in vitro* and *in vivo* PD-CSF-CTC cultures to produce a projection of how the transcriptome profiles were related (Figure 2A). For example, noncultured CSF contained multiple cell types, such as immune cells, fibroblasts, and melanocytes (CTCs), which were clustered in separate islands (Figure 2A). We were able to capture 148 CTCs and 149 CTCs, in the noncultured CSF of patient 9 and patient 12, respectively (Supplementary Figure 1). All *in vitro* and *in vivo* propagated PD-CSF-CTCs comprised entirely melanocytic cells. And as expected, there was heterogeneity between patients within the melanocytes cluster.

Next, focusing on the melanocyte (CSF-CTC) cluster stratified by individual patients (patients 9 and 12), we compared commonalities and differences in gene expression between noncultured CSF-CTCs, and *in vitro* and *in vivo* propagated PD-CSF-CTCs. Our scRNA-seq transcriptome data revealed approximately 20 000 expressed genes for all PD-CSF-CTC cultures. In the noncultured CSF-CTCs of patient 12, a total of 14 799 expressed genes were identified. However, we were only able to capture 6467 expressed genes in patient 9’s noncultured CSF-CTCs due to an RNA quality issue. (Figure 2B). Qualitative comparison of transcriptomes between noncultured CSF-CTCs and cells in cultures showed that the majority of genes were commonly expressed (Figure 2B). We found only 0.5% of identified genes were unique to noncultured condition. Among the expressed genes in noncultured CSF-CTCs from patient 9 and patient 12, there were 97.7% (6321) and 96% (14 200) of genes, respectively, retained after *in vivo* expansion. Overall, this suggests that *in vivo* inoculated PD-CSF-CTCs displayed a transcriptome resemblance to clinical samples, even after an *in vitro* culturing process.

### ScRNA-Seq Analysis Revealed Enriched Genes in CSF-CTCs From LMD Patients, Including IGF1R

**Figure 3.**
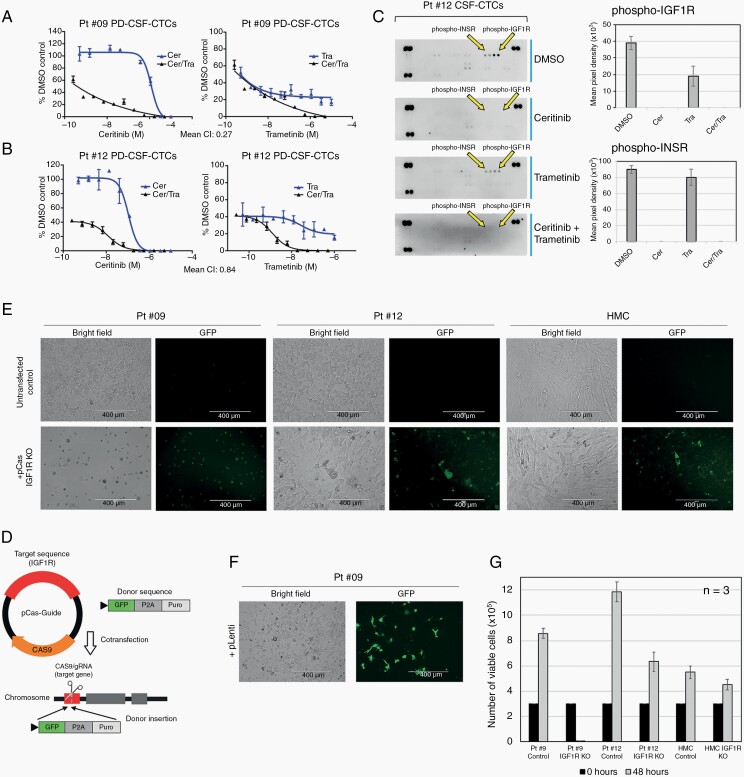
IGF1R inhibition promotes cell growth inhibition. (A & B) CellTiter-Glo Assay was used to assess the effects of ceritinib and trametinib on growth inhibition at 72 hours in patients 9 and 12 PD-CSF-CTCs. Synergy between (Mean CI) ceritinib and trametinib was analyzed where values < 1 indicated synergism, and >1 indicated antagonism. (C) Immunoblot from a Proteome-Profiler-Human-Phospho-RTK array for patient 12 PD-CSF-CTCs after 24 hours ceritinib, trametinib, or both treatments. DMSO was used as control. Arrows are pointing at duplicate spots representing activities of phospho-IGF1R and -INSR. Average pixel intensity was quantified using ImageJ Software. (D) Schematic diagram of CRISPR/Cas9-mediated *IGF1R* deletion, and the incorporation of GFP for positive selection. (E) Representative 20x bright field and fluorescent images of patients 9 and 12 PD-CSF-CTCs, and HMC after 24 hours of CRSPR/Cas9-guided *IGF1R* deletion. GFP was used a positive marker. Bar = 400 μm. (F) Representative 20x bright field and fluorescent images showing transfection of GFP into PD-CSF-CTCs using lentiviral vector did not induce cell death after 48 hours. G) Average number of viable patients 9 and 12 PD-CSF-CTCs, and HMCs after 48 hours of IGF1R depletion.

Leveraging the scRNA-seq data from patient 12, we next determine potentially clinically actionable targets. To do this, we refined the list of retained genes by screening patient 12’s scRNA-seq data against the transcriptomes of nontumorgenic cells (eg, immune cells and fibroblasts) found in this patient’s CSF, and filtered for melanocyte-specific gene signatures. After this process, we identified the melanocyte genes that were most enriched (average logFC in expression > 0.4) (Figure 2C, and Supplementary Table 3 and Figure 3A), which included *MLANA,*^19^*SOX9,*^20^*ErbB3,*^21^ and *IGF1R*^22,23^; this pattern is reminiscent of melanoma cells undergoing epithelial-mesenchymal transition,^20,24–26^ which can play key roles in tumor progression (Figure 2D). Furthermore, we extended our investigation examining the transcriptomes of noncultured CSF-CTCs from patients 8, 10, and 11 and found evidence that similar melanocytic gene signatures were enriched in these patients (Figure 2E). Interestingly, IGFBP2 expression was most enriched in CSF-CTCs that could be expanded *ex vivo* (Supplementary Figure 2). In patients 9’s and 12’s autopsy CNS specimens, we confirmed the presence of IGF1R activity in tumors (Figure 2F&G). Together, these data suggest that *in vitro* and *in vivo* propagated CSF-CTCs retained many of the biological pathways in melanoma, and these cells may be useful for functional analyses for LMD.

### Inhibition of IGF1R by Ceritinib Promotes Cell Growth Inhibition of PD-CSF-CTCs and Human BRAF V600E Melanoma Cells, Including Those That are Rendered Resistant to BRAF Inhibitors

To determine whether PD-CSF-CTCs could be used as a tool for functional analysis of therapeutic responses in LMD, as a proof of concept, we tested IGF1R as a drug target on the basis of our scRNA-seq data (Figure 2D). In addition, IGF1R was selected as a candidate because previously we found evidence of a correlation between LMD prognosis and IGF1 protein level in CSF.^2^ Because there are no FDA-approved IGF1R-specific inhibitors that have good blood-brain penetration ability, we decided to use ceritinib, a potent tyrosine kinase inhibitor that has been shown to work synergistically with trametinib (an FDA-approved MEK inhibitor) in preclinical wildtype melanoma models.^13,27^

Antiproliferative activities of ceritinib and trametinib were first validated in human *BRAF V600E* melanoma cell lines WM614 and the therapy (BRAF inhibition) resistant derivative, WM164R (Supplementary Figure 3A&B).^28^ Trametinib and ceritinib inhibited WM164 growth while only, as expected, ceritinib had an impact on WM164R. Of note, we observed ceritinib resensitized WM164R to trametinib (EC50 = 6.19e^–9^ M) (Supplementary Figure 3B) and both drugs acted synergistically in limiting the viability of both resistant and sensitive cell lines (mean CI, WM164R: 0.91, WM164: 0.27).

In PD-CSF-CTC cultures (patients 9 and 12) and briefly propagated CSF-CTCs (patients 9, 12, and 16), we also observed a high degree of sensitivity to varying degrees of ceritinib (Figure 3A&B and Supplementary Figure 3C). Trametinib was less effective, but similar to WM164 results and reports in other models,^13^ combination of trametinib with ceritinib proved synergistic with CI’s of 0.84 for patient 12’s and 0.27 for patient 9’s PD-CSF-CTCs (Figure 3A&B). Taken together, these data indicate that ceritinib may be a useful approach in treating MAPK inhibitor-resistant LMDs.

Activation of RTKs, such as IGF1R and INSR, are common mechanisms of resistance to MAPK-targeted therapy.^29^ Using an RTK array we found that high levels of IGF1R and INSR phospho-activities were reduced upon treatment with ceritinib (Figure 3C). To further determine the importance of IGF1R activity in LMD growth, we ablated *IGF1R* in PD-CSF-CTCs using CRISPR/Cas9. Successfully edited cells expressed green fluorescent protein (GFP), as the *IGF1R* cutting site was inserted with a *GFP-P2A* donor sequence (Figure 3D). Upon *IGF1R* knockout in PD-CSF-CTCs, we noted an increased number of detached cells after 24 hours and a significant decrease in cell viability after 48 hours compared to empty vector controls and control cell line (HMCs) (Figure 3E–G).

### Combined Ceritinib and Trametinib is Effective Against In Vivo Murine Xenograft Models of LMD

**Figure 4.**
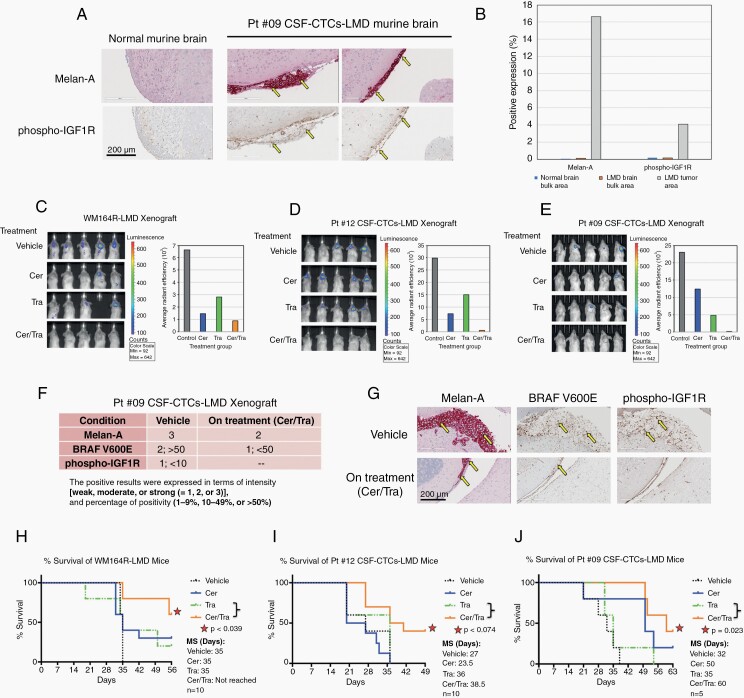
Ceritinib + trametinib treatment prolonged survival of melanoma-associated LMD *in vivo* in murine xenograft model. (A) Representative images of Melan-A and phospho-IGF1R staining of mice normal and LMD brains by IHC. LMD mice were generated using PD-CSF-CTCs. Bar = 200 μm. (B) Quantitative analysis of Melan-A and phospho-IGF1R expressions in the brain sections of (A). LMD in mice were generated by inoculating (C) WM164R, (D) patient 12 PD-CSF-CTCs, and (E) patient 9 PD-CSF-CTCs in CSF. Disease progression in each cohort was assessed by BLI, after three weeks of ceritinib, trametinib, or both oral therapies. (F) IHC scores of Melan-A, BRAF V600E, and phospho-IGF1R expression in LMD xenograft brain tissues after three weeks of ceritinib and trametinib treatment. (G) Representative IHC sections of (F); Bar = 200 μm. Survival graphs of (H) WM164R-LMD, (I) patient 12 PD-CSF-CTCs-LMD, and (J) patient 9 PD-CSF-CTCs-LMD mice after receiving ceritinib, trametinib, or both treatments were analyzed. Control mice received vehicle solution. Median survival (MS) was assessed. Survival graphs were compared statistically using the Mantel-Cox test, performed by GraphPad Prism 6 Software.

To test whether ceritinib and trametinib were effective in LMD xenografts (Supplementary Figure 4A), we first labeled PD-CSF-CTCs with a luciferase gene prior to injection into the CSF space of NSG mice, and used BLI to monitor growth over time. Without treatment, LMD mice survived for approximately 4 to 5 weeks. In *ex vivo* sections, we confirmed IGF1R activity to melanoma in the brain meninges by IHC (Figure 4A&B). These results were consistent with IHC for IGF1R in brain and spinal cord autopsy tissue sections derived from patients 9’s and 12’s (Figure 2F&G). Next, to test ceritinib and trametinib, we set up LMD cohorts composed of PD-CSF-CTCs (from patients 9 and 12), WM164R, and WM164 that were randomized into 4 groups; vehicle control, ceritinib, trametinib, or both. Using BLI as a readout of tumor growth we found that monotherapy treatment reduced tumor burden and the effect was more pronounced in the combination group (Figure 4C–E and Supplementary Figure 4B). Treated mice also showed a decreased IGF1R activity in brain meninges via IHC (Figure 4F&G). Interestingly, the efficacy of either ceritinib or trametinib varied between different LMD cohorts. For example, though neither of the monotherapy extended median survival for WM164R-LMD (Figure 4C) and patient 12’s CSF-CTCs-LMD (Figure 4D) cohorts, ceritinib monotherapy significantly enhanced median survival for patient 9’s CSF-CTCs-LMD cohort (*P* =.027), and trametinib was effective for WM164-LMD cohort (*P* = .003) (Figure 4E and Supplementary Figure 4C). However, and of note, survival was significantly improved when we combined both drugs for all LMD cohorts in this study underscoring the potential efficacy of these inhibitors for the treatment of LMD (Figure 4C–E and Supplementary Figure 4C).

Collectively, our results showed that CSF-CTC expansion was possible. Despite going through an *in vitro* and *in vivo* propagation processes, PD-CSF-CTC cultures maintained transcriptome resemblance to that of noncultured cells. These findings also provide support that PD-CSF-CTCs are impactful tools for better understanding LMD pathology testing the efficacy of targeted therapies.

## Discussion

LMD from melanoma is a devastating disease with a significant unmet medical need for effective treatments. The lack of PD-CSF-CTCs available for high-throughput drug screening for LMD has been a barrier to drug development. Here we showed the first successful propagation of CSF-CTCs *in vitro* and *in vivo* from patients with LMD from melanoma. These may be valuable assets to better understand the biology of LMD.

Successful establishment of CSF-CTCs was a trial-and-error process. We attempted a variety of culturing methods, such as fractional PB-CTC culture, growing CTCs in neurosphere and hypoxic conditions,^9^ or changing the media recipe after short culturing periods.^18^ None of the efforts proved effective, despite our experience with growing glioma stem cells.^30,31^ Ultimately, we successfully propagated CSF-CTCs using HMC-conditioned medium and supplemented with FGF and EGF.

There may be several reasons why CSF-CTCs are difficult to expand. Based on our experience, the likelihood of a successful CSF-CTC propagation relies on at least three elements. First, it is clear that standard *in vitro* culture conditions do not recapitulate the CSF microenvironment.^16^ The meninges secrete a variety of trophic factors/cytokines into the CSF (eg, FGF-2, VEGF-A, IGF, CXCL12, EGF, IGFBP2, and IGFBP6).^32–36^ Indeed, similar growth factors are present in HMC-conditioned media, which led us to speculate their importance to CSF-CTCs growth. For example, IGFBP2 modulates the bioactivity of IGF1/IGF1R, and is mainly synthesized in the choroid plexus and leptomeninges.^37^ It has been suggested that IGFBP2 levels in CSF may correlate with CNS malignancy.^38,39^ Similarly, IGF1 activity in melanoma LMD may determine disease prognosis.^2^

A second contributing factor to successful CTC establishment may be the relative scarcity of CTCs in patients’ CSF samples. Low number of CTCs from CSF posed a challenge when we attempted to develop a PDX model. The collection of CSF at autopsy allowed higher volumes of CSF to be collected (and hence more CTCs) than would be acceptable clinically. However, we may still require to briefly grow CTCs *ex vivo* for functional analyses. In our experiment, we successfully developed a CDX LMD model and we are aware that it may partially (but not fully) recapitulate the biological environment of LMD.^40^ We further posit the use of humanized mice may also facilitate high engraftment rates.^41^

Thirdly, it is likely that specific subset(s) of circulating CTCs are responsible for the development of LMD, and identifying these cells may allow for better enrichment of culture conditions, and successes in expansion in PDX models.^16,42^ Unfortunately, we did not have access to primary tumor specimens to perform scRNA-seq comparison with CSF-CTCs. We may be able to use this method to predict unique subpopulation of PD-CSF-CTCs that distinguished them from the primary tumor in future studies and be used to predict which melanoma patients will develop LMD.

As shown by our scRNA-seq data, *in vitro* and *in vivo* PD-CSF-CTC cultures were largely representative of CSF-CTCs before expansion. Though a small percentage (0.5%) of gene signatures were unique to noncultured CTCs in CSF, a majority of biological pathways were retained. We used this to select most commonly enriched pathway(s) shared between propagated and noncultured cells and test clinically relevant drug targets. For example, once we identified that IGF1R is enriched in CSF-CTCs, we confirmed that there was phopho-IGF1R activation in LMD samples from autopsy specimens and used IGF1R-depletion assays to confirm the requirement of growth. Hence, as a proof of concept, we attempted to target IGF1R activity in preclinical LMD model.

Because there are no FDA-approved/brain penetrant IGF1R inhibitors, we used ceritinib, a noncanonical drug against IGF1R as proof of principle and noted its effectiveness against melanoma cells in this LMD model with acquired resistance to MAPK inhibitors (as occurs clinically). Further functional and biochemical analyses to fully understand the mechanism of anti-IGF1R in the context of LMD are the focus of future studies. Whether ceritinib can be clinically used as an IGF1R inhibitor for patients with LMD is uncertain,^43^ as it is highly protein-bound in CSF^,44^ with only ~1.4% (0.012 μM) unbound drug. However, given the highly disrupted blood-CSF barrier found in LMD,^4^ effective dosages are completely unknown for therapeutic CSF penetration. We are currently assessing other inhibitors of IGF1R signaling to determine the potential of IGF1/IGF1R as drug candidates for LMD.^45,46^ Because *ErbB3* was also enriched in PD-CSF-CTCs, another strategy we explored is a dual inhibition of IGF1R and ErbB3. Since ErbB3 is sometimes regarded as “undruggable’ due to its lack of phosho-activity,^47^ allosteric inhibitors were recently developed to prevent ErbB3 from dimer signaling.^48^ In another study, bi-specific antibody for IGF1R and ErbB3 was tested in preclinical models of pancreatic cancer.^49^ Further investigation is currently underway in our lab to investigate ErbB3’s role in LMD.

We are aware of several limitations of our study. First, it is not clear how representative PD-CSF-CTC cultures are of the general population of patients with LMD from melanoma. A second limitation is somewhat conceptual: we could only generate long-term PD-CSF-CTC cultures from autopsies and, hence, this method cannot be used for “personalized LMD medicine.” If we can successfully optimize CSF-CTCs from Ommayas or LPs then we may be able to use these for individualized LMD treatment and identify, for example, the early emergence of treatment resistance and alter therapy. A third limitation is the quantity of cells per sample which limited by clinically acceptable volumes. This occurred in our scRNA-seq experiment where one CSF sample (patient 9) from an Ommaya had very small number of viable cells, which contributed to the RNA quality. This might reflect the fragile nature of CSF-CTCs and we will accumulate serial collection specimens when possible.

In conclusion, propagating CSF-CTCs from patients with melanoma-associated LMD may provide a valuable resource to better understand the biology of LMD and discover new therapeutic targets via strategies like high-throughput drug screening.

## Supplementary Material

noac054_suppl_Supplementary_Figure_S1

noac054_suppl_Supplementary_Figure_S2

noac054_suppl_Supplementary_Figure_S3

noac054_suppl_Supplementary_Figure_S4

noac054_suppl_Supplementary_Table_S1

noac054_suppl_Supplementary_Table_S2

noac054_suppl_Supplementary_Material

noac054_suppl_Supplementary_Legends
