# Corrigendum to: A preclinical model of patient-derived cerebrospinal fluid circulating tumor cells for experimental therapeutics in leptomeningeal disease from melanoma

**DOI:** 10.1093/neuonc/noac181

**Published:** 2022-07-29

This is a corrigendum to: Vincent Law, Zhihua Chen, Francesca Vena, Inna Smalley, Robert Macaulay, Brittany R Evernden, Nam Tran, Yolanda Pina, John Puskas, Gisela Caceres, Simon Bayle, Joseph Johnson, James K C Liu, Arnold Etame, Michael Vogelbaum, Paulo Rodriguez, Derek Duckett, Brian Czerniecki, Ann Chen, Keiran S M Smalley, Peter A Forsyth, A preclinical model of patient-derived cerebrospinal fluid circulating tumor cells for experimental therapeutics in leptomeningeal disease from melanoma, Neuro-Oncology, 2022, noac054, https://doi.org/10.1093/neuonc/noac054

In the originally published version of this manuscript, one of the two R21 numbers in the Funding section was incorrect; the numbers were given as R21 CA198550 and R21 CA216756. This has now been updated to R21 CA256289 and R21 CA216756.

In addition, in Figure 4, panel I was originally mislabelled as panel H.

These errors have been corrected.

