# Supplementary figures and images for: A preclinical model of patient-derived cerebrospinal fluid circulating tumor cells for experimental therapeutics in leptomeningeal disease from melanoma
Source: Neuro Oncol. 2022 Feb 25;24(10):1673–86. doi: 10.1093/neuonc/noac054 (PMC9527526; doi:10.1093/neuonc/noac054)

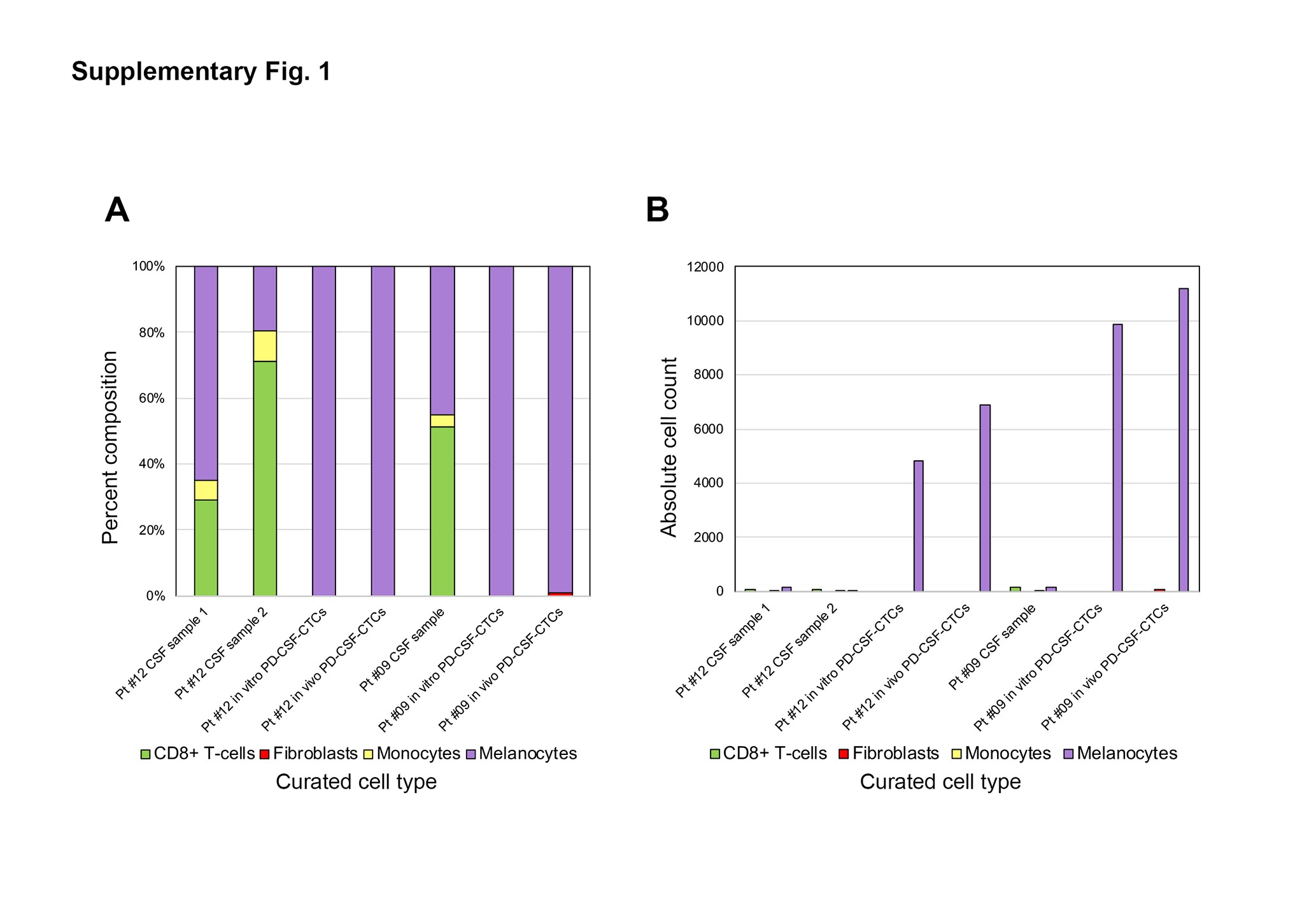

Supplement: noac054_suppl_Supplementary_Figure_S1 [file noac054_suppl_supplementary_figure_s1.jpeg]

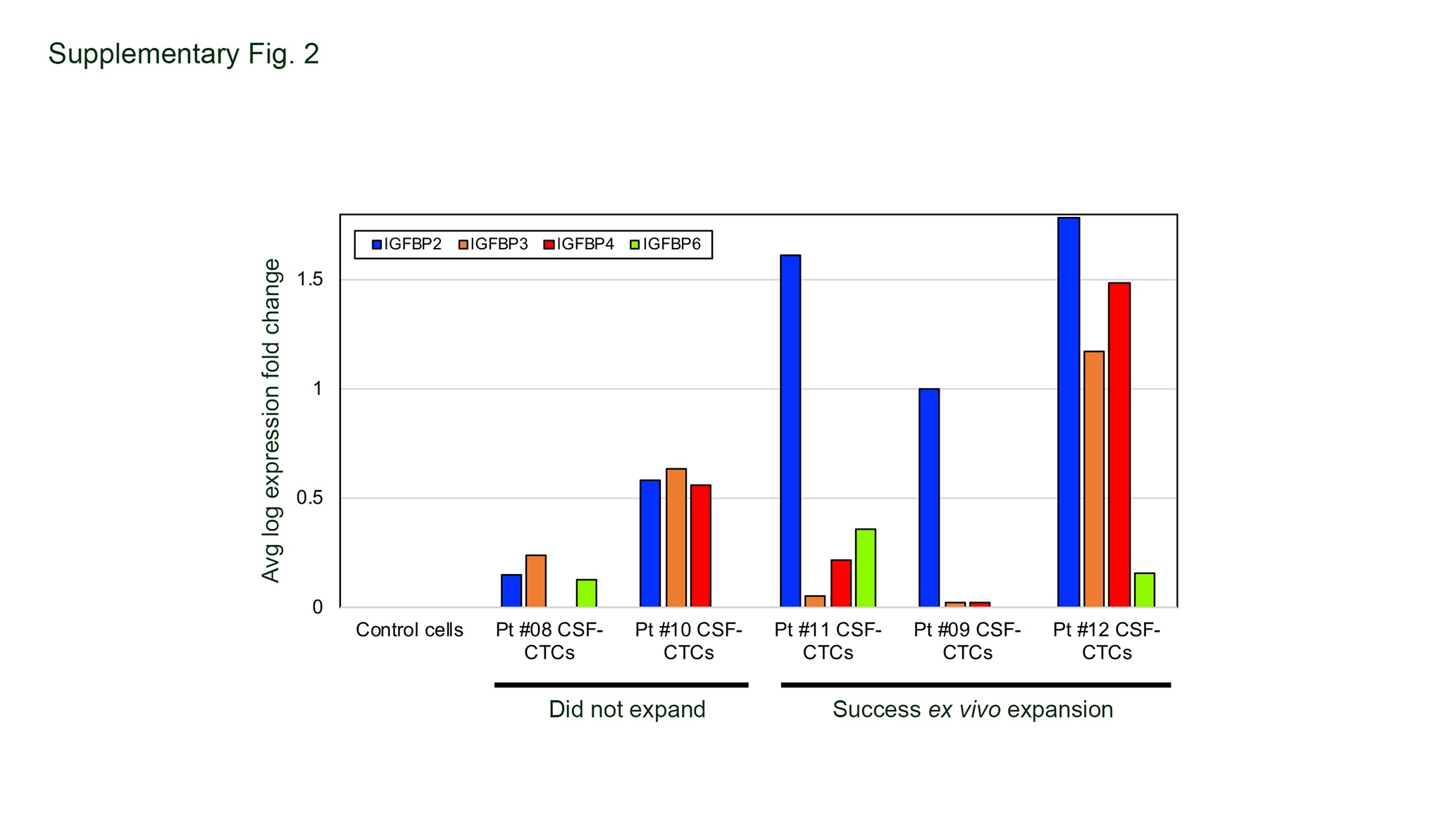

Supplement: noac054_suppl_Supplementary_Figure_S2 [file noac054_suppl_supplementary_figure_s2.jpeg]

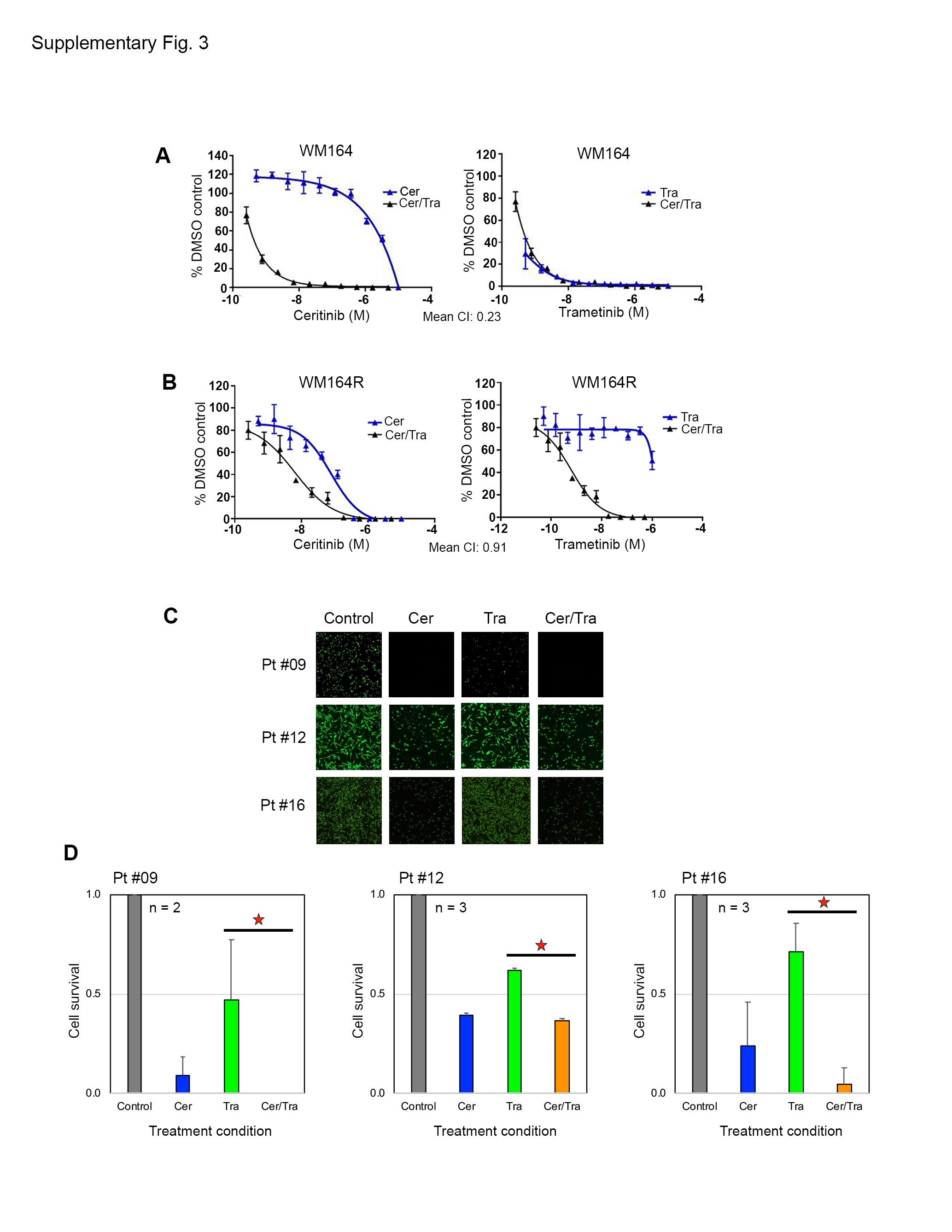

Supplement: noac054_suppl_Supplementary_Figure_S3 [file noac054_suppl_supplementary_figure_s3.jpeg]

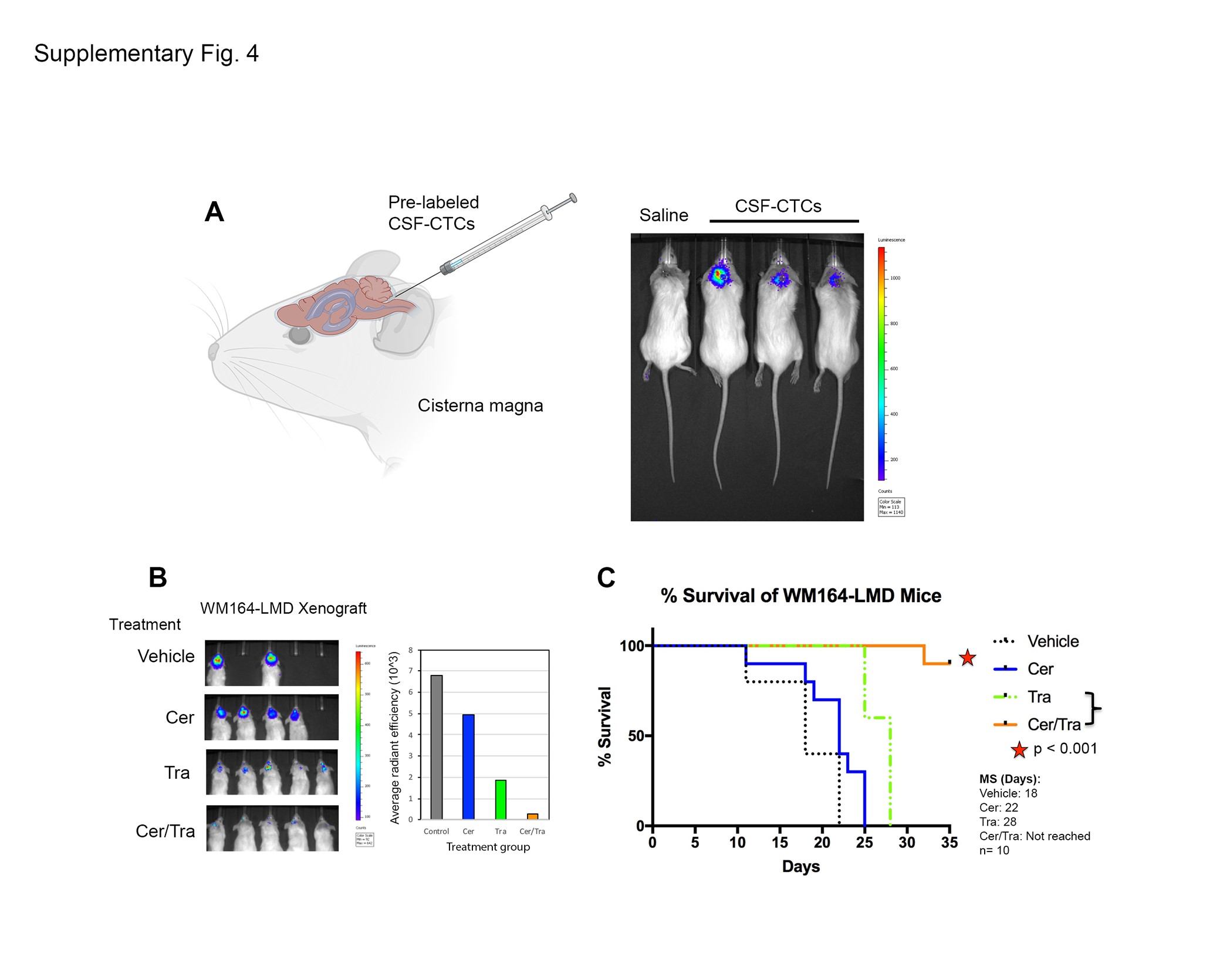

Supplement: noac054_suppl_Supplementary_Figure_S4 [file noac054_suppl_supplementary_figure_s4.jpeg]

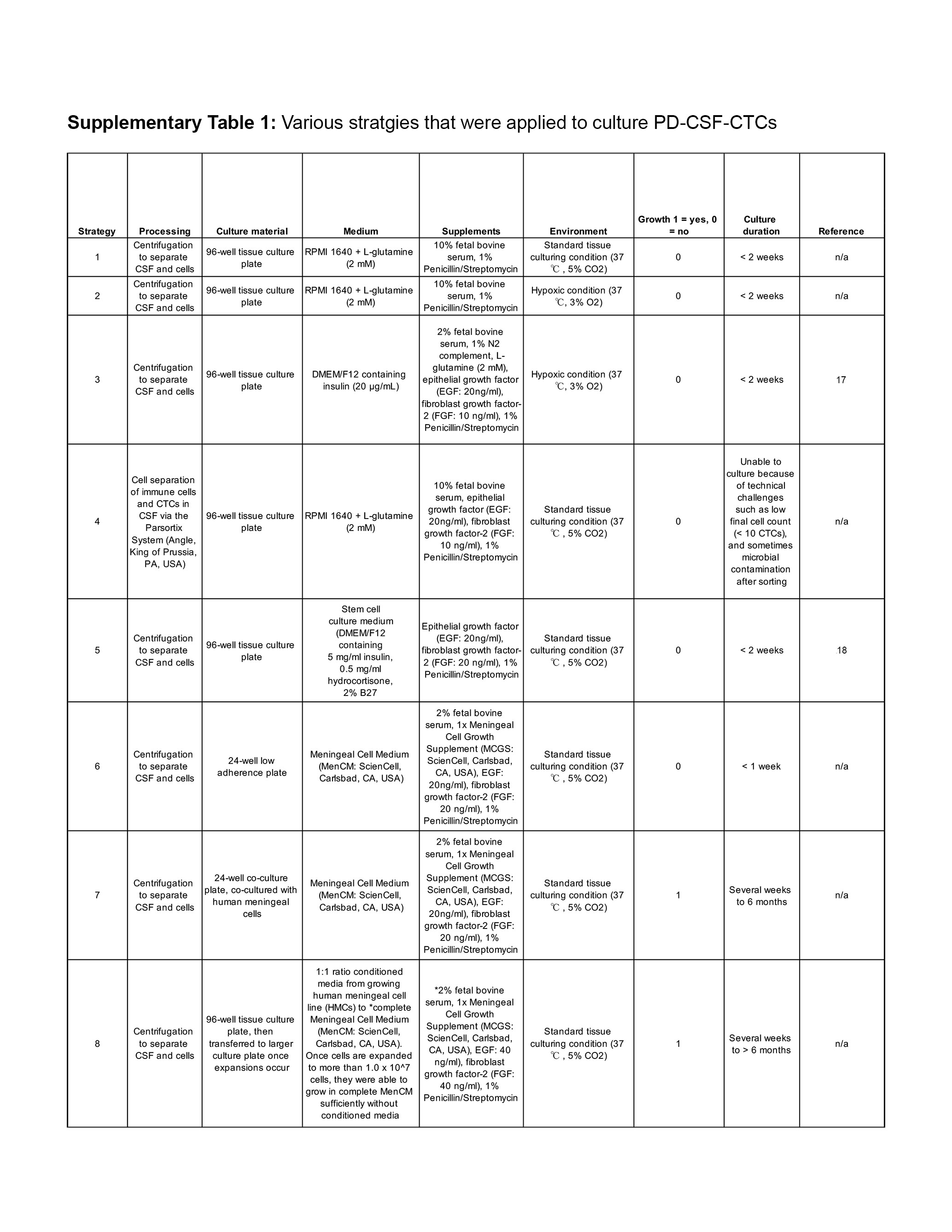

Supplement: noac054_suppl_Supplementary_Table_S1 [file noac054_suppl_supplementary_table_s1.jpeg]

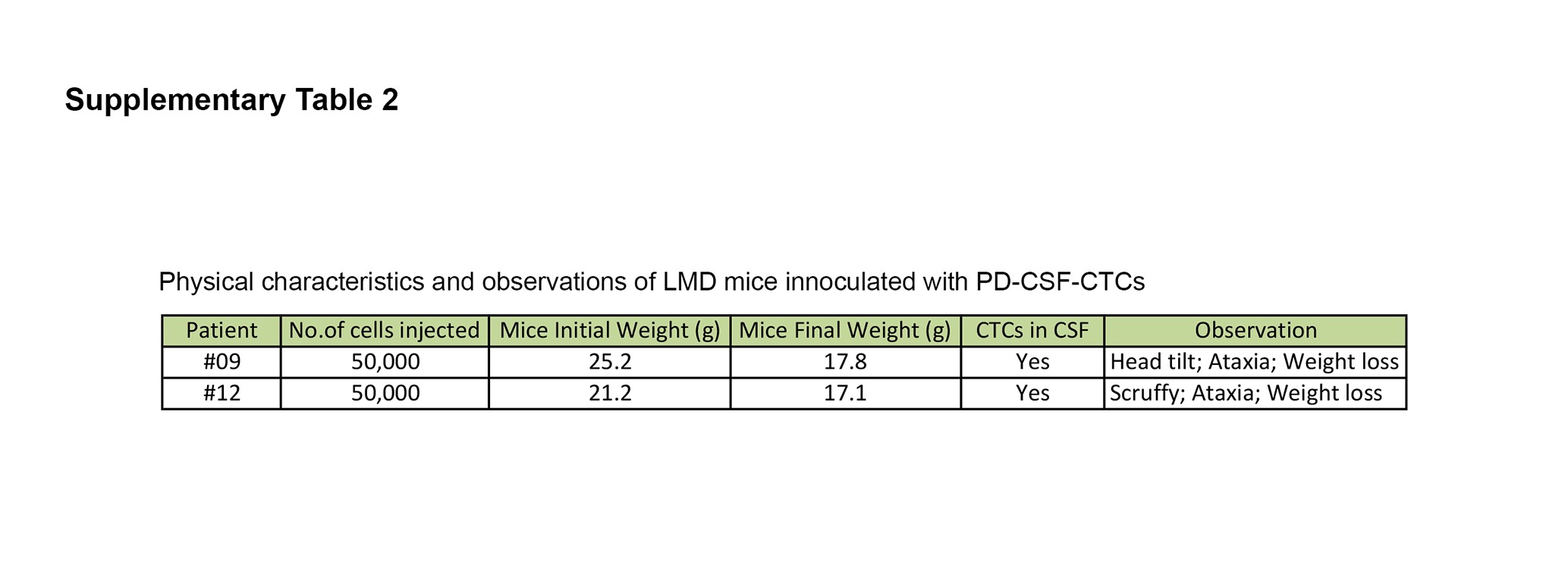

Supplement: noac054_suppl_Supplementary_Table_S2 [file noac054_suppl_supplementary_table_s2.jpeg]
