# Supplementary material for: A preclinical model of patient-derived cerebrospinal fluid circulating tumor cells for experimental therapeutics in leptomeningeal disease from melanoma
Source: Neuro Oncol. 2022 Feb 25;24(10):1673–86. doi: 10.1093/neuonc/noac054 (PMC9527526; doi:10.1093/neuonc/noac054)
Supplement: noac054_suppl_Supplementary_Material [file noac054_suppl_supplementary_material.docx]

**SUPPLEMENTARY METHODS**

***CellSearch for CSF-CTC enumeration***

Circulating melanoma cells were immunomagnetically captured with CD146 (melanoma cell adhesion molecule) and identified with specific anti–high molecular weight melanoma-associated antigens (MEL-PE) and 4′,6-diamidino-2-phenylindole (DAPI). Exclusion of endothelial cells (CD34–antigen-presenting cells) and leukocytes (CD45- antigen-presenting cells) were excluded using CD34- and CD45-specific antibodies respectively.

***Genotyping of PD-CSF-CTCs***

Genomic DNA extraction from *ex vivo* PD-CSF-CTCs was performed using the AllPrep DNA/RNA Mini Kit (Qiagen, Hilden, Germany) according to the product manual. Genomic DNA was submitted to Genewiz (South Plainfield, NJ) for single-nucleotide polymorphism genotyping/mutational analysis of exon 15 in the *BRAF* gene to determine whether PD-CSF-CTCs retained a *BRAF V600E* mutation after culturing.

***Melanoma cell line culturing***

Human melanoma cell lines WM164 and WM164R were from one of our collaborators, Dr. Keiran Smalley (H. Lee Moffitt Cancer Center and Research Institute, Tampa, FL, USA). The WM164R cell line was generated and maintained through chronic treatment with 2 μM of vemurafenib (PLX4032; Selleckchem, Houston, TX, USA). Both cell lines were cultured in Roswell Park Memorial Institute (RPMI) media (Corning Inc, Corning, NY, USA) with 5% FBS + 100 U/ml of penicillin streptomycin, with the addition of PLX4032 for the resistant cell line.

***Immunofluorescence assays***

Fixed cells were permeabilized with permeabilization buffer made with 0.4% Triton X-100 + 8% bovine serum albumin (BSA) in phosphate-buffered saline (PBS). Immunofluorescence staining was performed by incubating primary antibodies (1:250 dilution) overnight. The next day, the slides were incubated with immunofluorescence secondary antibodies (1:250 dilution) for 1 hour, and 300 nM 4’,6-diamidino-2-phenylindole (DAPI) (Thermo Fisher Scientific, Alachua, FL, USA) for 5 minutes. Washes were performed in between incubations using wash buffer (0.2% Triton X-100 + 0.2% BSA in PBS). Coverslips were mounted on glass slides with 3 μl each of Vectorshield (Biozol, Eching, Germany). Primary antibodies: anti-MLANA (Melan-A) and α-Smooth Muscle Actin (α-SMA), and secondary immunofluorescence antibodies were from Abcam, Cambridge, MA, USA. Cell images were captured using the Axio Imager Z2 (Zeiss, Oberkochen, Germany).

***RNA sequencing quality control and cell typing***

were performed using the R package Seurat to process the aggregated 20 069-cell transcript count matrix generated from the 10x Cell Ranger pipeline. Regularized negative binomial regression and a second linear regression against mitochondria read percentage, as implemented in SCTransform, were used to adjust for cell-to-cell technical variations. In parallel, we used multiple reference panels with the R package SingleR on the raw transcript counts to generate cell type candidates for each cell. Then we used the unsupervised clustering algorithm Louvain in the principal component analysis space to find cell groups among all cells that passed quality control. We assigned each cell group to a broad cell category using a combination of SingleR predictions, mostly via the BlueprintEncode panel, and literature review.

***RTK Array Quantification***

The RTK array immunoblot was pictured via chemiluminescence imaging using Odyssey Fc (LI-COR Biosciences, Lincoln, NE, USA). The pixel densities of the dots were quantified with LI-COR Odyssey Fc software, and the average signal (pixel density) of each duplicate spot was calculated for each slide. Phosphorylation (relative to 24 hrs with dimethyl sulfoxide [DMSO]) was quantified for each protein by subtracting the negative control dots within each panel.

***CRISPR/Cas9 assays***

CRISPR/Cas9 kits, which included two pCas-guided IGF1R-specific guide RNA (gRNA) vector clones and a donor sequence containing two markers (green fluorescent protein [GFP-P2A] and puromycin-resistant [Puro]) for positive selection, were purchased from OriGene Technologies (Rockville, MD, USA). The procedure was performed by following the manufacturer’s instructions. We decided to use green fluorescent expression as an indicator for edited cells.

***Immunohistochemistry and computer-assisted analyses***

Formalin-fixed paraffin-embedded tissue sections were evaluated for Melan-A, phospho-IGF1R, and BRAF V600E via immunohistochemistry (IHC) with optimized anti-Melan-A (Abcam, Cambridge, MA, USA), anti-phospho-IGF1R (Invitrogen, Waltham, MA, USA), and anti-BRAF V600E (Abcam, Cambridge, MA, USA) antibodies in the Tissue Core at H. Lee Moffitt Cancer Center and Research Institute. Slides were imaged with an Aperio AT2 slide scanner (Leica Biosystems, Buffalo Grove, IL, USA) with a 20×/0.7 NA lens. Images were imported into Visiopharm image analysis software version 2020.08. A thresholding algorithm was used to identify positive stain pixels for each biomarker. The thresholds for positivity were set by sampling negative stain regions to determine background intensity level. The area of each biomarker was calculated and exported to excel.

***Histopathologic scoring***

The assessment of tumor was scored as scant (1 [detectable only with careful examination]); patchy (2); or extensive (3). IHC results were scored as absent (0); weak (1 [requiring high powered examination to verify]); moderate (2 [cells within tumor deposits were categorized]); and strong (3 [intense brown staining]). The percentage of positive cells within tumor deposits was categorized as 1% to 9%, 10% to 49%, or above 50%.
