# Supplementary material for: A preclinical model of patient-derived cerebrospinal fluid circulating tumor cells for experimental therapeutics in leptomeningeal disease from melanoma
Source: Neuro Oncol. 2022 Feb 25;24(10):1673–86. doi: 10.1093/neuonc/noac054 (PMC9527526; doi:10.1093/neuonc/noac054)
Supplement: noac054_suppl_Supplementary_Legends [file noac054_suppl_supplementary_legends.docx]

**SUPPLEMENTARY FIGURE LEGENDS**

**Supplementary Table 1: Various CSF-CTCs culturing methods and strategies that were attempted**

**Supplementary Table 2: Physical characteristics and observations of LMD mice inoculated with PD-CSF-CTCs in cell line–derived xenograft model**

5.0 x 10^4^ patient 9 and 12 PD-CSF-CTCs were inoculated in NSG mice. Weights of mice before inoculation and at study end points were recorded. Physical observation was made during LMD progression.

**Supplementary Table 3: Lists of melanocytic genes and its fold changes in gene expression that were identified in non-cultured, and *in vitro* and *in vivo* cultured PD-CSF-CTCs of patient 12**

Melanocytic genes were mapped by screening against melanocytic profile and comparison to non-tumor cells found in CSF. Most commonly enriched melanocytic genes (log fold change (LogFC) in gene expression > 0.4) between non-cultured, and *in vitro* and *in vivo* cultured PD-CSF-CTCs were identified.

**Supplementary Fig. 1: Composition of cell population patients’ CSF samples and PD-CSF-CTC cultures**

**A)** Percent cell composition and **B)** Absolute number of cells identified based on cell type in each sample submitted for scRNA-seq analysis.

**Supplementary Fig. 2: Average expression of IGFBP subtypes in melanoma patients CSF-CTCs**

ScRNA-seq data analysis showing the average log expression of IGFBP2, 3, 4, and 6 in CSF-CTCs from patients 8, 9, 10, 11, and 12 compared to non-tumor cells (control).

**Supplementary Fig. 3: Ceritinib drug efficacy against melanoma cell line and PD-CSF-CTCs**

Ceritinib and trametinib drug efficacy against **A)** WM164 and **B)** WM164R. Synergy between ceritinib and trametinib was analyzed using the Chou-Talalay method. Mean CI calculations were performed by Compusyn Software where values < 1 indicated synergism, and >1 indicated antagonism. **C)** Ceritinib and trametinib efficacy non-CDX (short term culture) CSF-CTCs (patients 9, 12 and 16) after 72 hrs. Representative images of Calcein-AM live cell staining was shown. Live cells were stained in green. **D)** MTT assay showing viability of CSF-CTCs after 72 hrs of treatment.

**Supplementary Fig. 4: *In vivo* LMD xenograft model to test drug efficacies of ceritinib and**

**trametinib**

**A)** Schematic diagram luciferase-labeled cancer cells injection into the cisterna magna, rendering mice with LMD. **B)** Representative brain BLI images of WM164-LMD mice at third week of treatments, given either ceritinib, trametinib, or both. **C)** Overall survival of WM164-LMD cohort. Control mice received vehicle solution (saline). Median survival (MS) was assessed. Survival graph compared statistically using the Mantel-Cox test, performed by GraphPad Prism 6 Software.
